# An Adult Case of Bochdalek Hernia with Incarcerated Transverse Colon Successfully Treated by Hand-Assisted Laparoscopic Surgery

**DOI:** 10.70352/scrj.cr.25-0124

**Published:** 2025-06-10

**Authors:** Erica Nishimura, Momoka Oosawa, Kazuhiro Matsuo, Nozomi Watanobe, Risa Ohtani, Koshiro Matsunami, Takako Muroi, Asuka Hara, Keita Hayashi, Yuki Tajima, Yasushi Kaneko, Hiroto Fujisaki, Kumiko Hongo, Kikuo Yo, Kimiyasu Yoneyama, Kiminori Takano, Motohito Nakagawa

**Affiliations:** Department of Surgery, Hiratsuka City Hospital, Hiratsuka, Kanagawa, Japan

**Keywords:** diaphragmatic, hernias, laparoscopy

## Abstract

**INTRODUCTION:**

Bochdalek hernia (BH) is a congenital diaphragmatic hernia that is rare among adults. It is difficult to treat especially with an incarcerated abdominal organ. Although surgery via laparotomy or thoracotomy to repair the hernia with or without mesh reinforcement is the gold standard of treatment for BH, conversion to open surgery is performed to obtain a good surgical view and sufficient working space. Herein, we describe a rare case of BH with incarcerated distal transverse colon treated by hand-assisted laparoscopic surgery (HALS).

**CASE PRESENTATION:**

A 74-year-old man visited the outpatient clinic with the chief complaints of anorexia and abdominal distension. Contrast-enhanced computed tomography scan showed incarcerated left diaphragmatic hernia, with the distal transverse colon trapped in the left hemithorax with ischemic change. The patient was diagnosed with large bowel obstruction because of BH. Thus, emergency laparoscopic surgery was performed. However, it was converted to HALS because the colon could not be easily pulled back to the abdominal cavity. The transverse colon was carefully removed without perforation. The surgical course was uneventful, and the patient was discharged 1 week after surgery.

**CONCLUSIONS:**

HALS can be used when laparoscopic surgery is difficult.

## Abbreviations

BH
: Bochdalek hernia
HALS
: hand-assisted laparoscopic surgery

## INTRODUCTION

BH is a type of congenital diaphragmatic hernia that mainly occurs in infants due to failure of diaphragmatic closure. It is caused by failure of the posterolateral diaphragmatic foramina to fuse properly.^1)^ This disease is rare among adults, with a reported incidence of 0.17%–6%.^2)^ Surgery via laparotomy or thoracotomy to repair the hernia with or without mesh reinforcement is the gold standard of treatment for BH.^3,4)^ However, in some cases, conversion to open surgery is performed to obtain a good surgical view and sufficient working space. Herein, we report a rare case of BH in an adult treated by HALS.

## CASE PRESENTATION

**Fig. 1:**
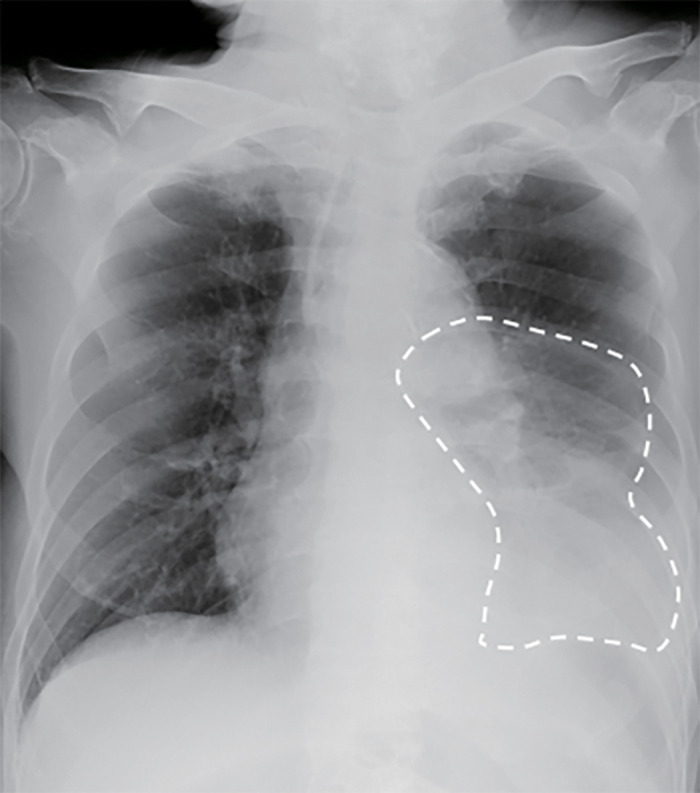
Chest X-ray showing an abnormal gas-filled mass in the left thoracic cavity.

**Fig. 2:**
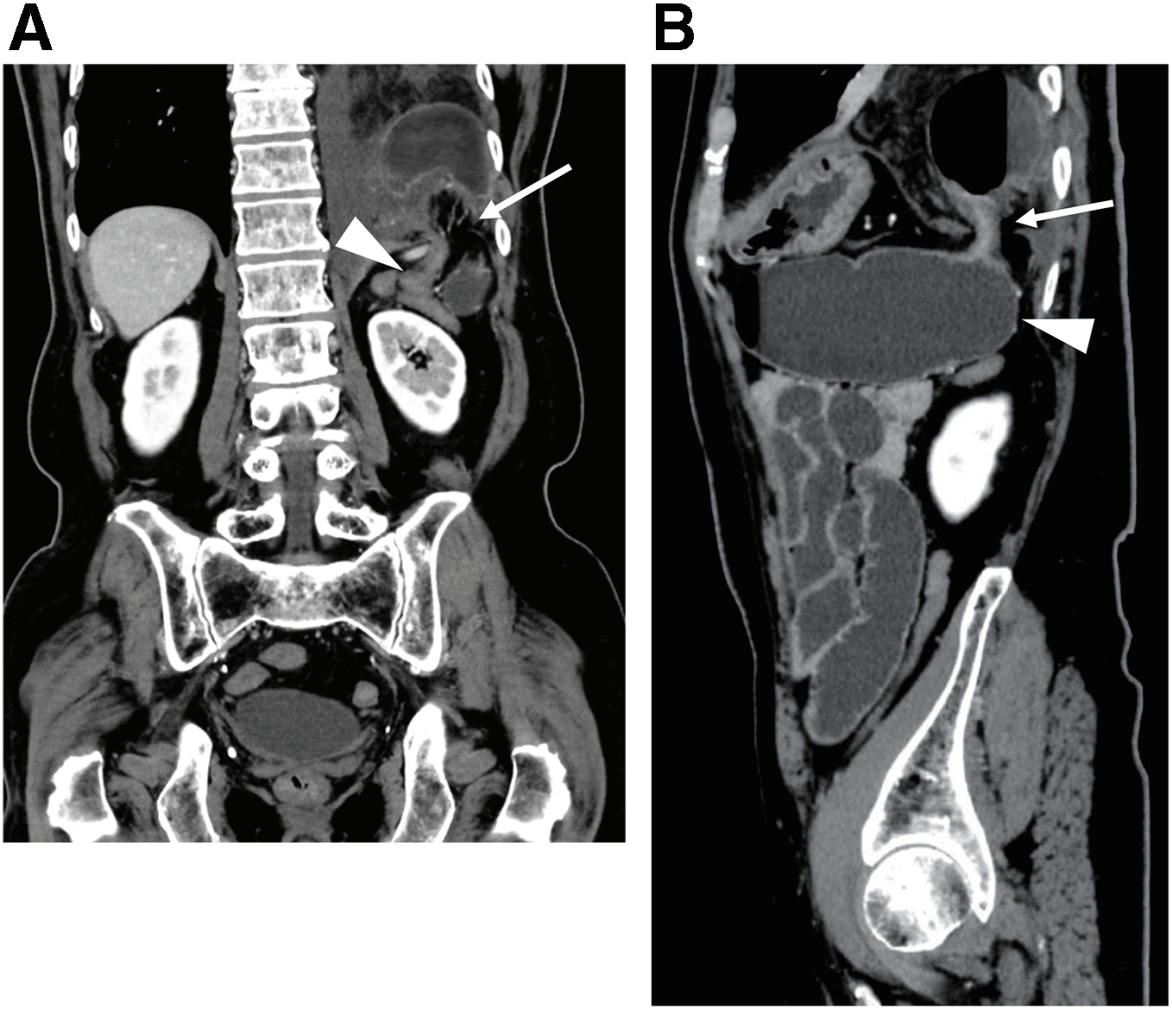
Computed tomography before surgery. (**A**) Coronal image showing a hernia defect (white arrow). The oral colon was dilated under the diaphragm (white arrow head). (**B**) Sagittal image showing the distal transverse colon herniating into the left thoracic space (white arrow).

A 74-year-old man presented to the outpatient clinic with chief complaints of nausea, anorexia, and abdominal distension, which gradually worsened 3 days prior to admission. He had no history of abdominal surgeries or trauma. His general examination was unremarkable. Although the abdomen showed mild bloating, it was soft with no severe pain. Laboratory tests showed leukocytosis and dehydration (**Table 1**). Although the CK level was within normal limits, the lactate level was slightly elevated. Meanwhile, chest X-ray revealed an abnormal gas-filled mass in the left thoracic cavity (**Fig. 1**), and contrast-enhanced CT scan showed incarcerated left diaphragmatic hernia with the distal transverse colon trapped in the left hemithorax. The oral side of the colon was dilated under the diaphragm (**Fig. 2**). The incarcerated bowel showed poor enhancement, bowel wall thickening, and edematous change. Giving the results of the blood tests with slightly elevated lactate with normal CK level, the incarcerated bowel was thought to be at the beginning of necrosis at this point. The patient was diagnosed with incarcerated large bowel obstruction because of BH. Thus, emergency laparoscopic surgery was performed.

**Table 1:** Laboratory tests on admission

Complete blood count		Blood chemistry	
WBC	19.8 × 10^3^/μL	AST	17 U/L
RBC	4.92 × 10^6^/µL	ALT	16 U/L
Hb	14.2 g/dL	LDH	185 U/L
Ht	4103%	ALP	85 U/L
Plt	298 × 10^3^/µL	γ-GTP	14 U/L
		BUN	84.0 mg/dL
		Cre	1.36 mg/dL
		Na	131 mEq/L
		K	4.4 mEq/L
		Cl	97 mEq/L
		CRP	8.83 mg/dL
		CK	133 IU/L
		Lactate	1.9 mmol/L

ALP, alkaline phosphatase; ALT, alanine aminotransferase; AST, aspartate aminotransferase; BUN, blood urea nitrogen; Cl, chloride; Cre, creatinine; CRP, C-reactive protein; Hb, hemoglobin; Ht, hematocrit; γ-GTP, gamma-glutamyl transpeptidase; K, potassium; LDH, lactate dehydrogenase; Na, sodium; Plt, platelet; RBC, red blood cell; WBC, white blood cell

**Fig. 3:**
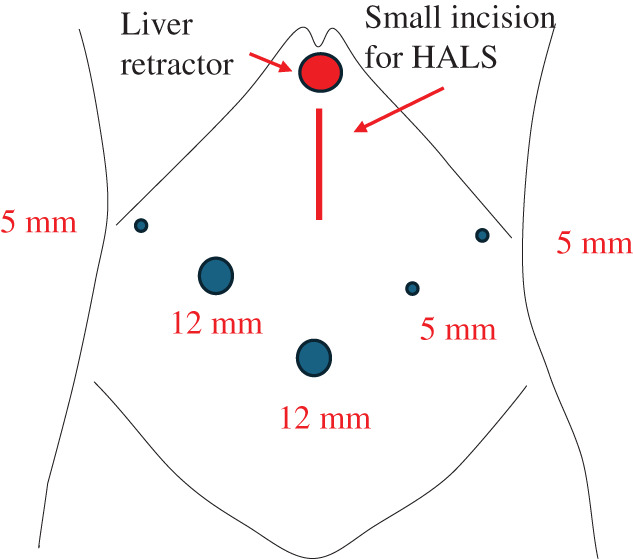
Port arrangement. A small incision was made for HALS. HALS, hand-assisted laparoscopic surgery

**Fig. 4:**
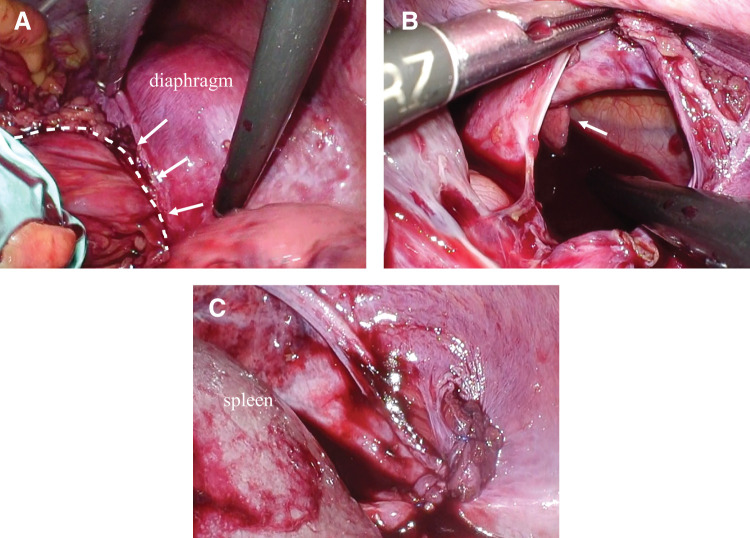
Surgical findings. (**A**) The incarcerated transverse colon (surrounded in the dotted line) was carefully pulled from the hernia orifice (white arrow) toward the abdominal cavity. (**B**) The size of the hernia orifice was 5 × 2.5 cm. The left lung was observed through the defect (white arrow). (**C**) The hernia defect was repaired using non-absorbable sutures.

The port arrangement is shown in **Fig. 3**. The surgical findings revealed incarcerated left diaphragmatic hernia containing the omentum and distal transverse colon. We attempted to return the contents of the hernia to the abdominal cavity using forceps. However, because of severe tension, it was difficult to return the colon. Moreover, we decided to convert to HALS because there was risk of colon rupture (**Fig. 4A**). A small incision, approximately 6 cm, was made on the upper abdomen, and an Alexis O-Ring (Applied Medical, Rancho Santa Margarita, CA, USA) wound retractor was inserted for a hand port. The adhesion of the hernia contents and orifice was loose, and the incarcerated organs were carefully pulled back to the abdominal cavity. The defect size was 5 × 2.5 cm, and the hernia orifice was smooth (**Fig. 4B**). The incarcerated organs were not resected because there was no sign of ischemic change. The hernia defect was closed using 3-0 nonabsorbable barbed sutures (V-Loc) (**Fig. 4C**). The total operation time was 155 min, and the intraoperative blood loss was low. The surgical course was uneventful. Oral intake began 2 days after surgery. He showed no symptoms of abdominal pain or respiratory discomfort and was discharged 1 week after surgery. Chest CT scan 2 weeks after surgery showed no recurrence of diaphragmatic hernia.

## DISCUSSION

Most cases of BH are diagnosed during the neonatal and postnatal periods.^5)^ In the adult population, most patients with BH are asymptomatic.^6)^ Moreover, the symptoms are vague and nonspecific, which leads to a high frequency of misdiagnosis.^3)^ According to Slesser et al., 38% of patients were misdiagnosed.^7)^ The delay of acute diagnoses may lead to strangulation, which may be life-threatening.^8)^ Although the patient in our case only had slight discomfort and nausea, the CT images showed ischemic change of the large bowel, which could cause high mortality if the diagnosis was delayed.

In the past few decades, the surgical approach to BH has shifted from open surgery to minimally invasive surgery. In their review of 184 cases of BH who underwent surgical intervention during 1955 and January 2015, Machado^3)^ reported that 40.3% of patients underwent laparotomy, whereas 27.7% patients underwent thoracotomy. Compared with thoracotomy, laparotomy may be more advantageous as it can provide good access to the diaphragm. Moreover, most general surgeons are more accustomed to laparotomy compared with thoracotomy.^1)^ However, surgeons might have difficulty in pulling back incarcerated organs, especially when they are strangulated. In the present case, there was a high possibility of empyema if the large bowel was perforated during the procedure because there was no hernia sac and the diaphragm had a congenital defect. HALS was extremely effective in placing the transverse colon back to the abdominal cavity because the surgeon could accurately feel the tension of the organs. Previously, two cases were converted to HALS to repair diaphragmatic hernia. First, Itamoto et al. reported a case of esophageal hiatal hernia after laparoscopic gastrectomy.^9)^ They used HALS because it was difficult to return the incarcerated transverse colon to the abdomen, similar to the present case. Second, Fukutomi et al. reported a case of BH with a previous history of abdominal incisional hernia repaired with mesh after hemicolectomy. HALS was effective in removing the adhesion around the hernia orifice and gastric corpus.^10)^ The postoperative course was uneventful in both cases. Taken together, HALS could be an alternative surgical approach to provide good visualization and minimal invasiveness when laparotomy is difficult.

The usage of mesh is still controversial. In general, mesh repair is usually used when the defect is too large to be primarily closed. Katsaros has published a systematic review of BH in the adult population.^1)^ In this review, a mesh was utilized in 47.8% of patients. On the other side, the recurrence rate after primary suture is reported to be 42%, which may be reduced by using a mesh.^11)^ However, there are some reports of complications that are specifically caused by the mesh. Erosion and infection of the mesh are both reported as severe complications, which demands surgery. Additionally, the mean defect size of the diaphragm was reported to be 7.01 ± 3.18 cm in this systematic review, which is larger than our case (5.0 cm). In this case, we repaired the orifice by simple closure without using mesh because there was no extensive tension after repair. In the future, we need to accumulate more cases and long-term follow-up data to develop a standard strategy to treat BH in adult cases.

## CONCLUSIONS

HALS was a safe and effective surgical approach to repair BH. HALS should be an option when minimally invasive surgery is difficult to proceed for BH.

## DECLARATIONS

### Funding

Not applicable.

### Authors’ contributions

EN wrote the initial draft.

All authors reviewed and approved the final version of the manuscript.

### Availability of data and materials

The data supporting the conclusions of this article are included within the article.

### Ethics approval and consent to participate

This study was conducted in accordance with the Helsinki Declaration of 2019 and later versions. Informed consent or a substitute was obtained from the patient for inclusion in the study. This work does not require ethical considerations or approval.

### Consent for publication

Informed consent was obtained from the patient for publication of this case report and accompanying images.

### Competing interests

The authors declare no conflicts of interest for this article.
